# *Feeling Under Pressure*: Perspectives of the Meaning of Love and Sexual Relationships Amongst Young Men in KwaZulu-Natal Province, South Africa

**DOI:** 10.1177/1557988319836632

**Published:** 2019-03-21

**Authors:** Thabang Manyaapelo, Sibusiso Sifunda, Robert A.C. Ruiter, Anam Nyembezi, Bart van den Borne, Priscilla Reddy

**Affiliations:** 1 Human Sciences Research Council, Population Health, Health Systems and Innovation, Pretoria, South Africa; 2 Human Sciences Research Council, HIV/AIDS, STIs and TB, Pretoria, Gauteng, South Africa; 3 Department of Work & Social Psychology, Maastricht University, Netherlands; 4 School of Public Health, University of the Western Cape, Cape Town, Western Cape, South Africa; 5 Department of Health Education & Health Promotion, Maastricht University, Netherlands; 6 Human Sciences Research Council, Population Health, Health Systems and Innovation, Cape Town, Western Cape, South Africa

**Keywords:** sexual context, HIV, men, romantic relationships, risky sex, multiple concurrent partnerships

## Abstract

This study aimed to explore perspectives on the meaning of love and sexual relations amongst young men in KwaZulu-Natal province of South Africa. Gaining insights into these perspectives will help to understand the sexual behaviors of these young men better and to eventually develop more effective HIV prevention interventions. Focus group discussions were conducted in two study areas using a predetermined semistructured discussion guide. The findings indicate that the phenomenon of romantic relationship try-outs together with the idea of “feeling under pressure” to propose love to more than one woman seem to be accepted practices that often lead to multiple concurrent sexual partners and therefore potentially risky sexual behaviors. The fear of impregnating a woman is seen to be of a more significant concern than acquiring a sexually transmitted infection due to the stigma and embarrassment associated with pregnancy outside marriage. Given these findings, it is recommended that future studies investigate perspectives on sexuality and reproductive health in male populations in great detail prior to the development of behavioral change interventions because failure to do so may hamper well-intended but poorly targeted health interventions.

The HIV and AIDS epidemic remains one of the leading public health priorities in sub-Saharan Africa despite more than two decades of efforts in an attempt to curb the spread. In 2016, the prevalence of HIV in the South African general population was estimated at 18.9% (UNAIDS, 2017), but varies between the different provinces with KwaZulu-Natal (KZN), the locale for this study, having the highest at 30% in the 15–49 age group (Shisana et al., 2014). Many interventions have been implemented across sub-Saharan Africa (Igumbor, Pengpid, & Obi, 2006; Kalichman et al., 2008; Kalichman, Cain, Eaton, Jooste, & Simbayi, 2011; Mathews et al., 2012; Morojele et al., 2014; Ousman et al., 2016; Sankoh, Arthur, Nyide, & Weston, 2014) and even though there is some shift in people’s beliefs, norms, and practices around issues of sexuality, romantic relationships, and overall sexual behavior, much more work still needs to be done in this area (Coates, Richter, & Caceres, 2008). Risky sexual behaviors such as inconsistent and incorrect condom use, engaging in sex while drunk or under the influence of illicit drugs as well as engaging in multiple concurrent partners continue to persist to cause new infections (Davis et al., 2016; Jama Shai, Jewkes, Levin, Dunkle, & Nduna, 2010; Turner et al., 2009; Yamanis et al., 2013). What is clear though is that interventions that promote male partner involvement in prevention of mother to child transmission (PMTCT) and those that promote adherence to antiretroviral therapy (ART) show better outcomes in morbidity and mortality (Kalichman, Cherry, White, et al., 2011; Mashaphu, Burns, Wyatt, & Vawda, 2018). Furthermore, interventions that seek to remove some of the structural barriers to testing result in more people testing overall and connecting more people to care programs. Other HIV/AIDS research in South Africa thus far has focused on gender inequalities and violence against women (Gibbs, Jewkes, Sikweyiya, & Willan, 2015; Jansen van Rensburg, 2007; Jewkes, Levin, & Penn-Kekana, 2003; Jewkes & Morrell, 2010; O’Sullivan, Harrison, Morrell, Monroe-Wise, & Kubeka, 2006; Sikweyiya, Jewkes, & Dunkle, 2014), and identifying social-cognitive determinants of risky sexual activities (Boer & Mashamba, 2007; Jemmott et al., 2007; Peltzer, Matseke, Mzolo, & Majaja, 2009; Tabana et al., 2012).

From as early as 2008, there has been a call to shift toward an approach that explores sexual context as a key factor in defining sexual relationships, love, and intimate partnerships in at-risk populations (Gupta, Parkhurst, Ogden, Aggleton, & Mahal, 2008). The sexual context is a multifaceted menagerie that incorporates structural, political, and economic as well as cultural influences on human behavior that need to be considered by interventions targeting risky sexual behavior (Parker, 2001). In the case of South Africa, a country in transition after a traumatic history of Apartheid and oppressive migratory labor practices, it is critical that social and sexual context be investigated to understand their contribution to the spread of HIV amongst local communities.

One example of the relevant social contexts is the association between the spread of HIV and migrants which has been well documented in the South African context (Lurie et al., 2003). This migration within South Africa was a result of repressive laws which forced African people to be confined mainly to rural areas (Kok, O’Donovan, Bouare, & van Zyl, 2003). This rural banishment could only be temporarily sustained since there was a growing demand for labor in the South African mines and factories. Migratory restrictions therefore partly contributed to the dismantling of African families where men were forced by circumstances to leave women to rear the children on their own in the rural areas (Reed, 2013). The contemporary sexual culture in South Africa is to a large extent influenced by this historical context (Leclerc-Madlala, 2002). It has been reported that decreased levels of social support and family separation have also been associated with HIV risk (Weine & Kashuba, 2012). This separation over extended periods had considerable influence on both men and women finding new sexual partners, thereby contributing to the phenomenon of multiple concurrent partnerships (MCP) which has been defined as overlapping partnerships where sexual intercourse with one partner occurs between two sexual acts of intercourse with another partner (Shumba, Mapfumo, & Chademana, 2011). This oscillating type of migration between urban and rural, between employment and homesteads, provided an enabling environment to the spread of HIV since the virus follows the movements of people (Lurie et al., 2003).

Some of the research examining the psychosocial determinants of risky sexual practices includes socio-contextual sexual practices, such as age-discrepant partners whereby young girls engage in sex with men who are much older than them, and more recently multiple concurrent sexual partnerships (Adimora, Schoenbach, & Doherty, 2007; Epstein & Morris, 2011; Evans et al., 2016; Mah & Halperin, 2010; Tibesigwa & Visser, 2015). These factors show an association with HIV infection rates and researchers agree that this relationship does not infer causality. More work is required to understand what motivates these sociocultural dynamics.

The HIV epidemic in South Africa is predominantly driven by heterosexual risk behaviors where multiple and concurrent partners are listed as one of the leading risk factors for transmission (Mah & Halperin, 2010). In South Africa where polygamy is practiced, it is not surprising that polygamous marriages and MCP are often conflated not taking into account that the type of marriage characterized by multiple partners is much more regulated by societies where this practice is common (Gausset, 2001). MCP among certain groups has been reported to be neither driven by a shortage of men nor an oversupply of women but by social factors such as lack of trust in partnerships and structural factors which are predominantly economic (Carey, Senn, Seward, & Vanable, 2010). This lack of understanding often influences current perceptions where a practice such as polygamy, which is cultural or context-specific, is viewed as something to be discouraged without an understanding of the meanings attached. As a result, interventions aimed at addressing MCP can be rendered ineffective due to poor understanding of contextual drivers.

Ogolsky, Surra, and Monk (2016) reported that a lot of the research about dating couples assumes that relationships are formed in the same manner across individuals. Meaning that the search for an ideal partner is the universal driving force, but the characteristics of what is defined as ideal differ across cultures (Buss et al., 1990). Buss et al. (1990) reported that characteristics such as chastity, good housekeeping, desire for home and children, education, and mutual attraction were not only influenced by cultural beliefs but also reported differences between men and women. The pivotal decision to choose one romantic partner over others should be viewed from the social-ecological framework with the premise that human development evolves through interactions with the social environment.

The prevailing societal norms will contextually guide behaviors and choices regarding romantic relationships. Exploration of the role of these norms in defining essential constructs such as love and sexual relationships is thus critical when thinking about ways to reduce the HIV/AIDS epidemic.

In this article, romantic relationships are defined as “mutually acknowledged ongoing voluntary interactions, commonly marked by expressions of affection and current or anticipated sexual behavior” (Collins, Welsh, & Furman, 2009, p. 632). In South Africa, contemporary notions of romantic relationships collide with more traditional lifeways and are influenced by migratory patterns between urban and rural locales. In KZN, for example, traditional practices such as *umhlonyane* and *umemulo* are still widely practiced in urban areas. Both these practices are rites of passage rituals for girls that involve a communal celebration that includes slaughtering of an animal to give thanks to the ancestors for the girl reaching her growth and maturity milestones. It would, therefore, be more informative if research into romantic relationships among the African population in South Africa is viewed in the context of these traditional practices which confirm the constant juxtaposition of urban and rural ideologies. Additional to this context is the systematically broken family units and the dysfunctionality arising from that (Holborn & Eddy, 2011). As much as the remnants of this complicated history bear heavily on the social and sexual contexts, they do not remove personal agency and choice. The very high levels of rape, intimate partner violence, and shifting masculinities are some of the examples of this social dysfunction (Hatcher, Colvin, Ndlovu, & Dworkin, 2014; Luyt, 2003; Lynch, Brouard, & Visser, 2010; Ratele, 2008; Wood & Jewkes, 1997; Wood, Lambert, & Jewkes, 2007). Coupled with this, research has also reported that young people choose to keep their sexual activities a secret for fear of being stigmatized, which impedes open discussions about topics related to sexuality and reproductive health (Harrison, 2008). It has been reported that young people wrestle with contemporary notions of romantic relationships. For example, material gain is believed to be more important for girls (Ranganathan et al., 2017), while boys prefer rural over urban girls since rural girls are perceived to be more conservative and still be virgins (Bhana & Pattman, 2011). In KZN, studies report that the dominant masculine perceptions were those of the male as a provider and MCP behavior being positively rewarded by the community for men and negatively for women (Hunter, 2004, 2005).

Given that the social and sexual contexts of romantic relationships in South African populations are associated with partner violence, long distance partnerships, the influence of traditional practices, and multiple concurrent partnerships, more research is still required to gain insight into how they are all interrelated. This study aimed to explore the meaning and understanding of love and sexual relations amongst young men in both the rural and the urban context and how these perspectives shape their perceptions about intimate romantic relationships as well as their notions on MCP and unplanned and unwanted pregnancies.

## Methods

### Study Setting and Sampling

This study formed part of a preliminary process of adapting a life skills intervention targeting men in two areas in KZN. The intervention was to target risky sexual behaviors such as no and inconsistent condom use, multiple partnerships, sexual activities when drunk, and also encourage supportive male roles in relationships. It was essential that this intervention was adapted for the local context not only regarding language but also in terms of being culturally sensitive. One location was a peri-urban area about 30 km from the city of Durban, while the other location was a deep rural village in the northern part of KZN, approximately 250 km from Durban. Four focus group discussions (FGD) were conducted, two in each study area. Each focus group consisted of 8 to 12 participants, with a total of 38 participants. The criteria for inclusion were males, between the ages of 18 and 35, isiZulu speaking, and the participants were required to be residents of the site under study. These discussions were conducted at community centers in the respective areas at a time most convenient for the participants. The FGDs were conducted using a predetermined semistructured discussion guide adapted from a previous study among soon to be released prison inmates in KZN and Mpumalanga provinces in South Africa (Sifunda et al., 2006).

### Data Collection

A purposeful sampling method was used where community leaders were instrumental in assisting in recruiting members of the community to participate in the FGDs. The discussions were organized around the following predetermined themes:

- Meaning and understanding of manhood in the community
- Meaning and understanding of love and sexual relationships
- Cultural norms and perceptions of love and sexual relationships
- Views on relationships, women, sexual partners, and fatherhood.
- Views on gender-based violence and interpersonal violence
- Views on multiple concurrent partners
- Perceptions of health risk including HIV/STDs among young men

The FGDs were conducted in community halls in the two selected study areas. Discussions were facilitated by two people, one interviewing while the other took notes. All FGDs were conducted in isiZulu by a team of trained isiZulu first language speakers. IsiZulu is the most widely spoken language in South Africa and is the language most spoken in the province of KZN. All focus groups were tape-recorded with digital recorders, after which the audio files were downloaded onto computers and then transcribed verbatim (Patton, 2002). Facilitators also kept field notes during the discussions for additional data gathering. Ongoing analysis of transcripts and interviewer field notes were utilized to determine saturation point of data collection. The discussions were transcribed by the same team and then translated into English. To ensure accuracy, the transcripts were back-translated into isiZulu by another team of isiZulu speaking research assistants, originally from the areas where the research was being conducted, who therefore were familiar with local dialects. The lead author and two of the coauthors are proficient in isiZulu and were responsible for monitoring the entire process of instrument development and data gathering.

### Data Analysis

The qualitative data management software ATLAS.ti (version 7) was utilized for coding the collected data. The method of analysis followed on the principles of transcendental realism, which recognizes that it is difficult for researchers to completely abandon any preexisting experiences or theories for fear of contamination; instead, the method encourages researchers to incorporate them. The interviews were analyzed by reading the transcripts multiple times in order to gain a sense of the flow of the discussion (Miles & Huberman, 1994). After that, each transcript was coded independently by three members of the research team. The first step of the analysis captured the manifest content of the interviews—the surface-level presentation of topics. Codes were independently identified then compared, and where similar, combined into single categories through consensus discussions. The second step produced subthemes or major categories that were inductively synthesized from the first step. Through a process of constant comparative analysis, relationships among the primary codes were integrated and condensed into the final emergent themes. This study received full ethical clearance from the South African Medical Association Research Ethics Committee (SAMAREC, Protocol MRC 1-09), which works according to the guideline of the Helsinki Declaration on ethical aspects in human experimentation. Additional permission was also granted by the local municipal offices and the traditional leadership in the areas concerned. Participants gave written informed consent to participate in the study by signing the consent form after it was sufficiently explained to them by the research team.

## Results

Four FGDs were conducted, two in the rural area and two in the peri-urban area. All the participants interviewed described themselves as heterosexual, and nearly all were unemployed. The rural participants were in the ages ranges of 18–25 (mean = 20.6) and 18–35 (mean = 24.4), while the peri-urban participants were in the age ranges of 18–25 (mean = 22.6) and 18–29 (mean = 23.8).

The peri-urban area is roughly 30 km from Durban with a majority African population. It is densely populated at about 12.95 km^2^ with approximately 31,600 households and only 32% of the residents were employed. The housing infrastructure is characterized by a mixture of free-standing dwellings, shacks, and hostels covering a small area (Statistics South Africa, 2003). The shacks and hostels are indicative of the migration that is common in South Africa between rural and urban areas. People come to the urban centers in search of employment opportunities, they usually end up living in informal settlements, and migrate a few times a year between urban and rural homesteads (Kok et al., 2003). The rural area is approximately 250 km from Durban, also with a majority African population. The rural area was approximately 8.66 km^2^ in size with about 178 households indicating a sparsely populated settlement. There was minimal road infrastructure in 2010–2011 when the data were collected and it made it hard to access all the men who could have been eligible to participate in the study.

Included in this section are the six themes from the analysis: meaning of love, sexual relationships, multiple concurrent partners, perceptions of manhood, perceived societal pressures, and lastly gender roles and division of labor. Together, all these factors interact to influence risky sexual behavior among the participants. In the analysis, the term “most” was used largely to describe consensus indicating that more than half of the participants shared the same sentiments. Since this study is qualitative in nature and it is not a representative sample of the population, it was decided not to use percentages.

### Meaning of Love

Despite the differences in life experience, age, and area of residence (rural and peri-urban), all the men interviewed explained that the foundations of romantic relationships for them is love. They described the isiZulu expression for when a man proposes love for a woman. The phrase is *ngiyakuthanda*, which literally means “I love you” as opposed to, for example, “I like you.” This expression is saved for and only used when a man wants to propose a romantic relationship to a woman. It is not used in a neutral context when a platonic relationship is proposed. Participants also described interesting notions of love such as emotional feelings of love interconnected with a physical attraction.

It’s the way we treat each other, although maybe it can be sex that we do right but we treat each other very special

Rural participant

There is this thing called random search it is where you scrutinise which girl you want by looking at the features. Some girls you will find they are beautiful but lack good manners.

Urban participant

Most participants mentioned that physical attributes of potential partners also played a vital role in defining the nature of the relationship and even the way that they will get treated. They emphasized qualities such as respect and good manners being key in their decision to start a romantic relationship. It was important for them to see that this person would not embarrass them before they could introduce them to friends or family.

With love we look at the qualities. Love is not about sex at all, you must not love a person because you had sex together.

Urban participant

Love is a deep feeling, it wakes you up in the early hours of the morning thinking about that person. That is the proof that you really love that person.

Rural participant

It was clear from the discussions that the participants could distinguish what they perceived to be casual relationships from what they thought could be potentially longer enduring commitments. There seemed to be some criteria they followed, although this appeared to be more subjective to the individual concerned. When it came to what the participants considered to be real love, there seemed to be a general consensus that there was a yearning to experience that.

### Sexual Relationships

Most participants expressed a level of distrust that they had against their partners in their respective relationships. This distrust seemed to be felt in their primary relationships as well as in the other relationships they had in multiple concurrent partnerships. The participants also had very strong perceptions that their partners did not trust them in turn. There was a general feeling among the participants that in their respective relationships they were aware of or suspicious of the existence of other sexual partners. It should be emphasized here that these views are from the men only and that the women were not interviewed.

I can say I do not love them equally because the other one stays very far from me, so that leads to the fact that I am not sure how she behaves where she is staying. So that makes me to be not sure as well about her because now there are diseases that can put me at risk, because I do know now as I am sitting here what she can be doing there.

Rural participant

Most participants expressed that as part of the preparation for marriage, they had to have more than one woman as a form of screening potential women for marriage at a later stage. This can be likened to having romantic relationship try-outs. The woman who somehow comes out as the best through this screening process will eventually be the one who is chosen to become a wife. The existence of other girlfriends is also seen as a way to create competition amongst girlfriends so that the winner eventually gets chosen to become a wife.

No, for me I can say I love my girlfriends although I don’t love them equally.

Urban participant

Maybe the one I love the most is the second one. In addition, the second one I can say she had moods, she was acting as if she is the one I love most. I ended up believing her the way she loves me but I had come to a situation where I decided to have another girl. The third one I was just trying to close the gap not because I love her but because I was trying to close the gap

Rural participant

For the participants who identified as having multiple romantic relationships, there did not seem to be a concerted effort to hide this behavior from their respective partners. There did not seem to be any fear of being found out nor was there a mention of any repercussions should the partners who didn’t know somehow find out.

I have four girlfriends but I do not have a child with any of them. Three of them are in the same school. The two know each other others don’t.

Rural participant

### Multiple Concurrent Partners

The majority of the participants described being involved in multiple concurrent sexual partnerships while only two participants spoke about having just one sexual partnership. The practice of MCP is driven by reasons some of which cultural and others based on individual choices. The participants who had described themselves as having several sexual partnerships mentioned that when they came across a nice woman *opakile* (sexy), it was usually difficult to resist the urge to speak to the woman or to leave her to pass by because they “feel under pressure” from physical attractiveness. This perceived “pressure” as experienced by the participants is what they gave as the reason for them to propose love to more than one woman at a time. The participants who practiced multiple partnerships described this pressure as being so overwhelming that they were unable to resist. The results of acting on this perceived pressure that they spoke of leads to acquiring more women as romantic partners even though they had existing romantic partners. None of the other participants disagreed with these sentiments.

When you are walking on the street and you come across an extremely attractive woman you will feel under pressure *ukuthi umshele* (initiate a romantic relationship). When we were growing up we were always told that *Intombi ayendlulwa* (you cannot pass a woman without complementing them on how nice they look)

Urban Participant /Rural participant

Participants who had multiple sexual partnerships alluded to certain idioms within their culture such as *Intombi ayaliwa* (dumping a woman is causing them bad luck). As a direct consequence of this widely held belief, participants mentioned that they often hang on to relationships that they may no longer be interested in nurturing just to avoid the situation of having to end a relationship with a woman. This belief may lead to the unintentional practice of multiple concurrent partners.

The way we were brought up we have always been told that *intombi ayaliwa* (dumping a woman is causing them bad luck) so that’s what makes it hard to end relationships sometimes, so the best thing is just to disappear and stop calling her.

Rural participant

Additionally, feelings of insecurity about their positions in these relationships may have led to multiple concurrent partnerships.

One of the things that make us have many partners is that we are uncertain about the relationship we are in.

Urban participant

### Perception of Manhood

All the participants mentioned the fear of being called *isishimane* (sissy) by their peers and friends in the community. *Isishimane* is an extremely derogatory term that is used to stigmatize men who are seen to struggle with initiating romantic relationships with women. The best approximate translation is the word “sissy.” It can be likened to calling a man “not man enough,” therefore viewed as one of the least favorable labels for a man to be called in this community. “Not man enough” implies that one is not assertive or aggressive enough as a man ought to be in pursuing women, therefore, making one a “nice guy” but with a very negative connotation attached. The nice guy connotation in this sense is seen as negative, because even though a man may be popular with women, if he does not act in a manner considered by his peers to be the required milestones in pursuing women, he would still be considered *isishimane*.

My problem is that in my family we are all boys and there are many of us. Therefore, I could not be the only *isishimane* in the family. We used to compete to see who can have the most girlfriends. So, if one brought a woman home all of us will be under pressure to match that and bring a woman the next time. We even had a “list” to compare numbers.

Urban participant

### Perceived Societal Expectations

All the participants reported very strong underlying cultural values and expectations regarding the importance of the ability to have children within a marriage. This expectation is seen to put a lot of pressure on women to prove their fertility to their partners before getting married and thus can lead to women intentionally getting pregnant to prove that they can bear children.

You know what I can say is that the girls in this area they do not want to use condoms. I went to the other girl last night, and I was carrying condoms and when I tried to ask her that let us use condoms she refused she did not want anything to do with condoms. I said let us use condoms and she said no, I explained that they are helping not to get pregnant do you know that, but then she did not want to listen. I did not use the condoms and that worries me a lot, how I will be able to handle this.

Rural participant

Despite the apparent modernization amongst indigenous communities, certain cultural practices related to love, sex, and marriage are still widely practiced and adhered to. Pregnancies before marriage are still widely frowned upon as evident in the continued existence of the payment of *inhlawulo* (damages) which is a fine for impregnating an unmarried young woman and is usually associated with shame and stigma amongst community members and is viewed as an embarrassment to both the boy and the girl as well as their families.

My parents have had to pay *inhlawulo* (damages) for all the three girls that I have made pregnant because I am still studying.

Rural participant

Furthermore, most of the participants expressed that some of the women “trick” them into impregnating them by saying they were on contraceptives when in reality they were not. This perceived deception was spoken about with very strong emotions because it seems that the young men’s reason for using condoms was solely to avoid unwanted pregnancies. Participants expressed that what typically happened was that they would as a couple use condoms in the initial stages of the relationship but once the woman confirmed that she was on some form of contraception the couple would in agreement stop using condoms. If the woman had lied and it turned out that she was not using any pregnancy prevention method, the couple would face an unwanted pregnancy. There were no disagreements from the other participants.

After I got a job of being a driver I got a lot of girls but I make sure that I use a condom. I started to use the condom since 1998 until today I have a girlfriend of five years she always insists that we should go and test (so as to stop using condoms). I explained to her that I can’t because since I did testis operation I might not be able to make kids.

Urban participant

### Gender Roles and Division of Labor

Historically, one of the critical milestones signifying a transition from a boy into manhood was the ability to accumulate resources that can assist to first pay *lobola* (bride price) for the intended wife and secondly provide a home for the future family. Men who were unable to fulfil these roles were traditionally viewed negatively. In the current study, there was an overwhelming sense of entitlement and ownership among the participants when it came to matters of *lobola*. Most participants felt that them paying *lobola* inherently bestowed upon them the authority to dictate the terms in their respective relationships and that any acquiescing when it came to couple decisions would emasculate them. The fact that they were in charge should not only be known by their partner but by the community in general so that their manhood could be asserted. None of the other participants disagreed with what was being said.

If I am the one who pays *lobola* (bride price) then my word should be final as in the (participant says his surname) household as the man I am in charge

Urban participant

Participants also alluded to sometimes being ridiculed by community members of both genders when they did certain caring acts in their relationships that should typically be viewed in a more positive light. This somehow leads to males assuming the role of a negative masculine type in order to avoid being taunted for displaying what should usually be viewed as positive and healthy in a loving relationship. An example is when men accompany their female partners to the clinic for reproductive health purposes but get ridiculed for doing so. This is generally a very positive supportive role that should be encouraged, but the participants said that the taunting resulted in them stopping with this behavior.

Sometimes when you try to treat your woman well by accompanying her to the clinic everyone will start saying you are trying to be a “nice guy.” Even the nurses at the clinic will make fun of you and not allow you to come with your girlfriend into the consultation room

Urban participant

It was revealed in the interviews that some of the participants did not live with both parents and alluded to the fact that their communities were not as closely knit as before.

Our fathers are not here they go and work in Johannesburg, so most people that are at home are the women they do not have time to stay together and discuss their problems.

Rural participant

## Discussion

This study aimed to get a better understanding of how young men view their romantic relationships and their perspectives on the meaning of love within these relationships. The study was part of a preliminary process that sought to develop and adapt a health behavior change intervention to help address risky sexual behaviors contributing to HIV transmission in KZN. Given the history of oppression and forced labor migration in South Africa coupled with some of the highest incidences of sexual violence against women in the world, it is imperative that we learn more about men’s perspectives on their relationships with women.

Romantic love in relationships is often characterized by the desire to be understood, respected, supported, and trusted by the partner one is in this relationship with (Smith, Nunley, & Martin, 2013). These are just some of the qualities that people seeking companionship would hope to enjoy. However, the expression of love interests varies across cultures where in the Western context there is a dating period where the word “like” is mostly used to be followed, after some time, by the word “love” should this relationship “get more serious” (Ackerman, Griskevicius, & Li, 2011). More recently though there is a call to adopt “romantic realism” in an attempt to foster a more pragmatic approach to the modern realities which contribute to high divorce rates globally (de Botton, n.d.).

In the population under study, the declaration of love does not have any accompanying verbal prelude as seen from the participants. This confession of love does not automatically imply a commitment to the woman pursued as the young men also talk intensely about this phenomenon labeled as romantic relationship try-outs in this study. Although it seems ubiquitous among the young men that this test phase is necessary, it does not appear that it is communicated to the intended romantic partner. These try-outs open the door to more problems. Since culture dictates that it is bad luck to dump a woman, the participants find themselves trying out more than one romantic interest at any given time and therefore partly explaining the phenomenon of multiple concurrent sexual partners. MCP is further exacerbated by the perceptions from peers that one is a sissy if he has not yet started to engage in sexual activities or has not initiated romantic interests at a determined time in his life. As if the situation is not primed enough for risky behaviors, there is also the perception of “feeling under pressure,” which can be viewed as another justification for acting recklessly. Although previous studies report that dominant and prevailing masculinity norms contribute to risky sexual behavior (Harrison, O’Sullivan, Hoffman, Dolezal, & Morrell, 2006), there is still a need to investigate the context of sexual interaction in more detail. Future research needs to elaborate more on the determinants and influences impacting on these gender roles to explain why men think that such behavior is acceptable.

This phenomenon of romantic relationship try-outs may have some affinity to the concept of *isoka. Isoka* has been described as a term that was used to refer to an unmarried man who was popular among girls and engaged with multiple partners (Hunter, 2004, 2005). Although *isoka* is explained as being prominent in the earlier part of the 20th century, there was evidence of a decline and a shift in how it was perceived by both women and men with the rise in *isoka lamanyala*, which means an undesirable form of *isoka* who played with multiple women without any intention of marrying any of them. This is because marriage along with certain initiation processes were seen as a rite of passage into manhood where a man would be expected to have the responsibilities of looking after his wife and children. The rise in HIV infections is believed to have played a role in this decline and change in perceptions about the term *isoka*. It should be highlighted that none of the participants from the current study mentioned the concept of *isoka* at any time during the FGDs. Given that this present study was conducted nearly 10 years after the seminal work by Hunter (2005, 2006), it is possible that this concept could have evolved considerably. Future studies should explore the connection between polygamy and this more contemporary notion of romantic relationship try-outs. Is it possible that the principles guiding polygamy could have evolved first to create notions of *isoka* then later culminated into the new phenomenon of romantic relationship try-outs? The latter is characterized by taking on multiple partners without any intention of marriage but now perceived as normal because there is no social disparagement.

Certain terminology and use of language such as *isishimane* (sissy) may inadvertently lead to young people taking certain decisions due to the poor understanding of the context and origins of those words. The common misconception is that this word is used to induce peer pressure from other men to force men to engage in MCP, but the hard reality is that even women can use this label on men to get them to act in a certain way. This study demonstrates that young men in these communities sometimes engage in harmful relationship practices as a result of the various societal pressures that they may experience, such as the notion of a “nice guy” is viewed as a negative attribute. Some authors describe *isishimane* to be the opposite of *isoka* wherein the former is characterized by the inability to attract a single lover while the latter is the ability to play with multiple partners as explained earlier (Hunter, 2004, 2005; Leclerc-Madlala, 2002).

The idea that paying *lobola* (bride price) bestows certain entitlements such as dictating the terms of a romantic relationship was observed among the participants. This is however not a surprising finding since previous research emphasizes the pivotal position of *lobola* in a man’s life (Hunter, 2005). A man, therefore, can only be considered a real man by his ability to gather resources which can afford him to take a wife. This entitlement could also open a door for men to dictate unsafe sexual practices like refusing to use condoms as reported in previous studies (Campbell, Nair, Maimane, & Gibbs, 2008). In certain instances, it is the women themselves who feel disempowered to assert themselves in romantic relationships because *lobola* was paid for them (Cornman et al., 2011).

What was very interesting was the finding that fear of getting a woman pregnant was a much bigger concern to the participants than the potential of getting infected with HIV or other STIs. As a result, young men stop using condoms once they feel that the risk of impregnating a girl has been eliminated. The cultural conundrum that unfolds in this present study is this: while the young men are still undecided about which woman they will eventually choose to marry, these same young men expect to engage in these romantic relationship try-outs with some of the women. On the other hand, the young women are expected to show that they are capable of bearing children yet they are not supposed to fall pregnant outside a committed relationship that will lead toward marriage.

A similar study investigating the contextual influences of relationships in young people in a KZN rural area reported that it was young men who were more likely than young women to engage in multiple partner behaviors (Harrison, Cleland, & Frohlich, 2008). Most importantly, the authors discovered that these women were aware that their male partners had other romantic relationships. This means that if a behavioral intervention were to be developed for this community targeting risky sexual practices, it would have to address issues of multiple concurrent partnerships differently from conventional approaches. Before the development of this intervention, researchers will need to fully understand why men, for example, feel they cannot end relationships with their romantic partners, but most importantly investigators need to comprehend what makes it acceptable in that society for men to engage in multiple sexual partnerships. Are these factors which promote MCP purely cultural or are they exacerbated by the contemporary urban context of better access to resources and the easier digital accessibility of potential sexual partners? The present study highlights certain social norms that have evolved whereby the stigma of an unwanted pregnancy carries more weight than acquiring an STI and where for men having multiple romantic partnerships seems acceptable.

In another earlier study in Mpumalanga province of South Africa, it was reported that women were more likely not to use any contraception until after the first birth (Tollman, Kahn, Collins, & Ngwenya, 2001). The investigators alluded to the lack of access to health facilities as a possible reason for this. The expectations to show childbearing capabilities as found in the present study could be another factor that explains this phenomenon. Therefore, a limited understanding of sexual context can severely hamper well-intended health behavior interventions. Similar to the findings of the present study, a recent study emphasizes the importance of designing interventions that have a specific focus on the contextual level factors when investigating HIV, STI, and risky sexual behavior as this may assist to strengthen the evidence for causality (Ward-Peterson et al., 2018).

Some of the participants also alluded to fathers being absent due to work commitments much farther from the homestead. This father absence was due to the historical legacies of Apartheid’s separate race group spatial planning that still has a lot significance on the present-day context. Africans were designated to live in the rural parts of the country and were forced to commute into the cities for work. It has been reported that childhood and adolescent development is positively enhanced by both parents participating in the upbringing of their children. In the absence of a father, the differences can be seen in development where girls exhibit an earlier onset of menarche (Guardia, Nelson, & Lertora, 2014) and in boys there can be behavioral problems such delinquency (Simmons, Steinberg, Frick, & Cauffman, 2018) or other risky behaviors such a substance use and multiple sexual partnerships (Alleyne-Green, Grinnell-Davis, Clark, Quinn, & Cryer-Coupet, 2016). The perceptions of love and sexual relationships as expressed by the participants of this study may be strongly influenced by the absence of parents. Future research needs to investigate absent parents more closely in relation to the findings in this study and perhaps compare views from young men in absent parent homes versus present parent homes. A better understanding of how family dynamics affect perception of love and sexual relations can assist to develop more precise behavioral interventions against risky sexual behaviors.

Current global HIV prevention efforts use a three-arm model which is made up of the biomedical, structural, and behavioral interventions. These interventions include voluntary male circumcision, prevention from mother to child transmission, curbing risky behaviors, and tackling some of the environmental, economic, and socio-political issues which leave people vulnerable to HIV infection (UNAIDS, 2016). In sub-Saharan Africa specifically, there are interventions to increase ART adherence (Kalichman, Cherry, Kalichman, et al., 2011), to reduce HIV stigma in PMTCT (Peltzer et al., 2018), to link HIV infected people to care (Mavegam, Pharr, Cruz, & Ezeanolue, 2017), school-based interventions to prevent STIs and HIV (Sani, Abraham, Denford, & Ball, 2016), and interventions that target the reduction of alcohol and drug use which in turn lead to risky sexual behaviors (Carney et al., 2018; Wechsberg et al., 2013). As much as all these interventions contribute to the fight against HIV, more interventions focusing on sexual context need to be developed. The findings of the present study contribute to a better understanding of sexual context and how sexual relationships are perceived among young men in KZN. It is in a thorough understanding of the sexual context that behavioral interventions can be better designed for the intended communities while taking cognizance of the cultural sensitivities.

## Limitations

The findings reflect the views and beliefs of a relatively small sample of young men from one specific region of South Africa and therefore should be interpreted with caution. A purposeful sampling method was used due to structural challenges such as the vast distances between villages and the lack of transport for the community members. The research team could only access community members who were available. This resulted in FGDs with young men who were mainly unemployed or not in school. When interpreting these results, it should also be considered that the nature of FGD can also be limiting. Given that the participants reside in the same area and possibly have an overlap in terms of circles of friends and close acquaintances, the views expressed may possibly have been sanitized.

Despite these limitations, this study was still able to elicit views and perceptions about sexual content not previously reported on as far as this study was able to ascertain. Romantic relationship try-outs with many partners, the idea of “feeling under pressure” together with the notion of being culturally restricted to dump girlfriends are findings that help to explain the phenomenon of multiple sexual partner concurrency further. This information will assist in the development of health behavior interventions to practice safer sexual behaviors.
